# Dispersion Estimation and Its Effect on Test Performance in RNA-seq Data Analysis: A Simulation-Based Comparison of Methods

**DOI:** 10.1371/journal.pone.0081415

**Published:** 2013-12-09

**Authors:** William Michael Landau, Peng Liu

**Affiliations:** Department of Statistics, Iowa State University, Ames, Iowa, United States of America; The University of Chicago, United States of America

## Abstract

A central goal of RNA sequencing (RNA-seq) experiments is to detect differentially expressed genes. In the ubiquitous negative binomial model for RNA-seq data, each gene is given a dispersion parameter, and correctly estimating these dispersion parameters is vital to detecting differential expression. Since the dispersions control the variances of the gene counts, underestimation may lead to false discovery, while overestimation may lower the rate of true detection. After briefly reviewing several popular dispersion estimation methods, this article describes a simulation study that compares them in terms of point estimation and the effect on the performance of tests for differential expression. The methods that maximize the test performance are the ones that use a moderate degree of dispersion shrinkage: the DSS, Tagwise wqCML, and Tagwise APL. In practical RNA-seq data analysis, we recommend using one of these moderate-shrinkage methods with the QLShrink test in QuasiSeq R package.

## Introduction

In the last five years, groundbreaking new RNA sequencing (RNA-seq) technologies have considerably improved studies in genetics that previously relied on microarray technologies. RNA-seq technologies have several advantages over microarrays, including less noise, higher throughput, and the power to detect novel promoters, isoforms, allele-specific expression, and a wider range of expression levels. So it is not surprising that RNA-seq has become ubiquitous in experiments that investigate the regulation of gene expression across different conditions, such as levels of a treatment factor, genotypes, environmental conditions, and developmental stages.

In a typical RNA-seq experiment, reverse transcription and fragmentation convert each RNA sample into a library of complementary DNA (cDNA) fragments, or tags. Next, a sequencing platform, such as the Illumina Genome Analyzer, Applied Biosystems SOLiD, Pacific Biosciences SMRT, or Roche 454 Life Sciences, amplifies and sequences the tags. After sequencing, a subsequence within each tag, called a read, is recorded. After the resulting collection of reads, or library, is assembled, the reads are mapped to genes in the original organism’s genome. The number of reads in a library mapped to a gene represents the relative abundance of that gene in the library. The investigator typically assembles all the read counts of multiple libraries into a table with rows to indicate genes and columns to indicate libraries. Please consult references by Oshlack, Robinson, and Young [1] and by Wang, Li, and Brutnell [2] for details regarding sequencing technologies, gene mapping, and data preprocessing.

A central goal of RNA-seq experiments is to detect genes that are differentially expressed : i.e., ones for which the average number of reads differs significantly across treatment groups. Improving the detection of differentially expressed genes opens new ways to control organisms at the molecular level, advancing fields like agriculture engineering, personalized medicine, and the treatment of cancers, contributing to social welfare.

Some of the most popular new statistical methods that detect differentially expressed genes from RNA-seq data rely on the negative binomial (NB) probability distribution. If a random variable, 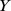, has an NB(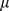, 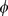) distribution –i.e., a negative binomial distribution with mean parameter 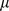 and dispersion parameter 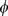 – then the probability mass function (pmf), expected value, and variance of 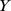 are:
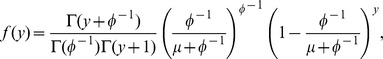


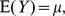


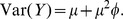



Cameron and Trivedi [3] show that as 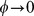, 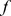 converges to the pmf of the Poisson(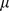) distribution, a distribution with mean and variance both equal to 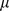. So the dispersion parameter, 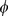, is a measure of the extra variance of 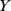 that the Poisson(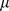) distribution does not account for.

In an RNA-seq dataset, the number of reads, 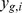, mapped to gene 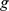 in library 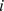 is treated as a random draw from an NB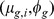 distribution. Here, 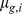 is the unnormalized mean count of gene 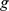 in library 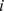, and 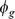 is the gene-wise (“tagwise”) dispersion assigned to gene 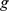. In addition, the model assumes that 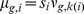, where 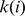 is the treatment group of library 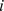, 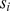 is the normalization factor of library 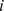, and 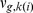 is the normalized mean count for gene 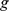 in each library of treatment group 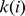.

It is common practice to include in the model the library-wise normalization factors, 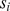, because counts in an RNA-seq data table may differ significantly across treatment levels for reasons other than the differential expression of genes. For instance, different RNA samples may be sequenced to different depths, making the libraries vary in size (total number of reads per library). To account for a possible variation in sequencing depth and other factors that may cause variation in library size, each column is assigned a normalization factor, 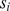, to be used in later analyses. There are several choices for the normalization factors. For instance, according to Si and Liu [4], taking each 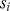 to be the 0.75 quantile of the counts in library 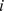 is a simple method that performs relatively well. Another popular option is the method proposed by Anders and Huber [5], which divides each count by the geometric mean count of the corresponding gene and then takes the medians of the these scaled counts within each library. The Trimmed Mean of M Values (TMM) method by Robinson and Oshlack computes each normalization factor from the trimmed mean of the gene-wise log fold changes of the current library to a reference library [6].

With the above preliminaries taken care of, we now turn to the main issue of this article: the estimation of the dispersion parameters, 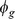. Each 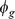 is a measure of the extra variance, relative to the Poisson (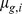) distribution, of the read counts of gene 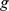. Since they control the variances of the counts, these 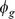
’s play an important role in hypothesis tests that detect differentially expressed genes. Underestimating a dispersion parameter is equivalent to underestimating the variance relative to the mean, which may generate false evidence that a gene is differentially expressed. Inversely, overestimating a dispersion may cause a truly differentially expressed gene to go undetected. For the sake of accurately detecting differentially expressed genes, it is important to choose an effective method for estimating dispersion parameters.

First, we briefly review several popular dispersion estimation methods (implemented in freely-available R-language packages, AMAP.Seq, DSS, edgeR, and DESeq). We also touch on some popular tests for differential expression. Next, using a simulation study that draws fundamental information from real datasets, we compare the practical effectiveness of the methods in terms of the accuracy and precision of the point estimates and the effect on the performance of tests for differential expression. In the results and discussion sections, we discuss the distinguishing features of the most successful dispersion estimation methods.

Studies by Wu, Wang, and Wu [7] and by Yu, Huber, and Vitek [8] also include simulation-based comparisons of methods for estimating negative binomial dispersions from RNA-seq. However, the authors of these studies were primarily concerned with inventing and validating their own methods. Since we do not propose any new methods here, our study has less personal bias than otherwise. In addition, our comparison is broader in scope. Only three methods were compared in the article by Wu, Wang, and Wu, six were compared in the study by Yu, Huber, and Vitek, and both studies ignored alternate versions of these methods. On the other hand, we consider alternate versions of five classes of methods (for example, the “Common”, “Tagwise” and “Trended” versions of the APL method described later), giving us a total of ten methods to compare. Considering alternate versions not only broadens the comparison, but also helps us isolate key features that help the good methods succeed. Lastly, our scheme for simulating datasets from the negative binomial model is based on real data and preserves observed relationships between the dispersions and the gene-specific mean counts. On the other hand, in the study by Wu, Wang, and Wu, dispersion parameters were simulated independently from the means. Yu, Huber, and Vitek use several simulation schemes and real datasets, but none of their realistic schemes simulates the negative binomial model, and according to these authors, the rest of the simulations favor either sSeq (their method) or DESeq (described later).

## Methods

### Existing Methods

#### Dispersion estimation methods

In RNA-seq data analysis, we could apply methods based on counts for each gene separately to estimate model parameters, such as the Quasi-Likelihood (QL) method reviewed below. However, in RNA-seq data, there are typically tens of thousands of genes, but only a few counts per gene with which to estimate gene-specific parameters, a typical example of a “large 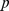 small 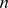
” scenario. In such cases, methods based on each gene separately are sub-optimal because they do not make use of most information contained in the whole dataset. Several methods proposed recently try to improve dispersion parameter estimation by using more information from the dataset. We review them after introducing the QL method.

#### The Quasi-Likelihood (QL) method

The QL method estimates a dispersion parameter independently for each gene. This method [9] [4], implemented in the AMAP.Seq R package by Si and Liu, iteratively estimates the mean and dispersion as follows:

- The MLE, 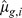, of each 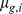 is obtained by maximizing the negative binomial log likelihood given 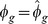 and the read count, 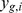.
- The tagwise dispersion estimate, 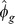, given 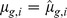, is computed via the quasi-likelihood (QL) technique reviewed by Robinson and Smyth [10].

The QL method estimates tens of thousands of dispersion parameters, but uses only a few read counts to compute each estimate. More sophisticated techniques make use of a larger number of read counts to estimate each 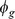. Specifically, they borrow information across genes and shrink the 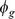
’s towards a common center. Each of the next four dispersion estimation methods applies some form of shrinkage.

#### The Weighted Quantile-Adjusted Conditional Maximum Likelihood (wqCML) method

As explained by Robinson and Smyth [11], the wqCML method shrinks the dispersions towards a common value using a weighted likelihood approach. The method first calculates pseudo-data from the original data with a quantile-adjustment procedure so that all the library sizes become equal. Next, the method estimates each 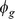 by maximizing the weighted likelihood, 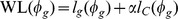. Here, 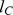 is the “common” log likelihood, the negative binomial log likelihood under the restriction that all genes share the same dispersion value, and 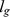 is the negative binomial log likelihood on the pseudo-data conditioned on the sum of the pseudo-counts of gene 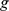. The tuning parameter, 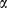, represents the extent that the method “shrinks” individual tagwise dispersions towards the single dispersion given by the common likelihood. In practice, 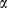 is calculated with the empirical Bayes rule described by Robinson and Smyth [11].

The wqCML method is implemented in the R package, edgeR, designed by Robinson, McCarthy, and Smyth and available at bioconductor.org [12], [13]. The user can choose between estimateTagwiseDisp(), which shrinks each dispersions towards a common estimate via wqCML, and estimateCommonDisp(), which estimates a single dispersion for all the genes by maximizing 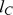.

#### The Cox-Reid Adjusted Profile Likelihood (APL) method

The wqCML method only applies to completely randomized designs with two treatment groups. In the APL method, McCarthy, Chen, and Smyth [14] extend wqCML’s idea of shrinkage via weighted likelihoods to the framework of generalized linear models, which can handle more complex designs, potentially with multiple treatment factors and/or blocking factors. McCarthy, Chen, and Smyth [14] apply the loglinear negative binomial model given by 
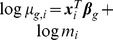
, where 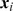 is a vector of covariate values specifying the experimental conditions on library 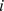 (i.e., 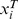 is the 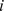
’th row of the design matrix), 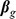 is the vector of experimental design parameters corresponding to gene 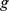, and 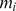 is the total number of reads in library 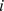.

To estimate the 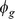
’s, McCarthy, Chen, and Smyth [14] make use of the tagwise Cox-Reid adjusted profile likelihoods given by the loglinear model above instead of the ordinary negative binomial likelihoods in the wqCML method. The authors describe three different variations on the APL method, which use these adjusted profile likelihoods in different ways to achieve three different kinds of dispersion shrinkage. The “Common” variation sets all the dispersion estimates equal to the common value that maximizes the arithmetic mean of the APLs over all genes. The “Trended” variation, which estimates a different dispersion for each gene using adjusted profile likelihoods while modeling the 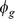
’s as smooth functions of the gene-wise average read counts, heavily shrinks the dispersion estimates toward a common trend. The “Tagwise” variation shrinks each gene’s dispersion estimate towards the common dispersion estimate of a set of neighboring genes. McCarthy, Chen, and Smyth [14] implement this method in the edgeR R package with the functions, estimateGLMCommonDisp(), estimateGLMTrendedDisp(), and estimateGLMTagwiseDisp().

#### The Differential Expression for Sequence Count Data (DESeq) method

Like the wqCML and APL methods, the DESeq method by Anders and Huber [5] borrows information across genes to shrink the dispersion parameters. DESeq differs from the other methods mainly in that it uses directly normalized read counts and makes more use of the observed variance-mean relationship in the data.

Anders and Huber [5] reparameterize the negative binomial model in terms of the mean and variance, and further parameterize the variance in terms of the mean, normalization factor 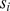, and a new “raw variance parameter”. They then use the normalized counts, 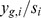, to compute the normalized negative binomial means and raw variance parameters for each gene-treatment group combination. The dispersions are calculated directly from these means and raw variance parameters.

As with the APL method, there are three variations on the DESeq method giving different ways to shrink the dispersions. The no-shrinkage variation transforms the estimated raw variance parameters directly into the estimated gene-wise dispersions without any shrinkage. The “Trended” variation performs a regression of the raw variance parameter estimates on the estimated means, and then computes the estimated dispersions from the fitted values on the trend. (In the DESeq R package, the implementation of the DESeq method, the user can choose between local and parametric regression to compute this trend. However, the parametric regression in the package is prone to failure and leads to poor point estimation test performance in our simulation study. Hence, only the local regression results are presented in this article.) The “Maximum” variation computes the maximum of each raw variance parameter estimate and its fitted value on the trend and then computes the dispersion estimates from these maxima. This last method of dispersion shrinkage is conservative, allowing overestimation of the dispersions, but guarding against underestimation in order to avoid false positives in tests for differential expression. The DESeq method is implemented in the R package, DESeq, available at bioconductor.org.

#### The Dispersion Shrinkage for Sequencing (DSS) method

The Bayesian paradigm more naturally accommodates the notions of “borrowing information” and “shrinkage” than the Frequentist paradigm. Hence, the DSS method by Wu, Wang, and Wu [7], an empirical Bayes approach, is particularly elegant. Rather than explicitly force the dispersions to shrink to a common value or trend as in the wqCML, APL, and DESeq methods, the DSS method seamlessly incorporates shrinkage with a Gamma-Poisson hierarchical model in which the dispersion estimates naturally shrink towards a common log-normal prior. The hierarchical model in the DSS method is
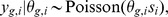


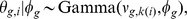


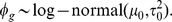



Here, the gamma distribution is parameterized in terms of its mean, 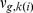, and dispersion, 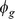, where 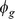 is the reciprocal of the shape parameter. The marginal distribution of 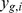 is NB(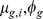), where 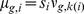 as before. Each dispersion estimate is taken to be the mode of the conditional distribution of 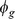 given 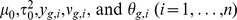. An adapted method of moments technique is used to estimate 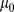 and 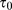. Wu, Wang, and Wu [7] implement this method in the R package, DSS, available at bioconductor.org.

#### Methods of testing for differential expression

With a model specified and all the parameters estimated, we can test for the differential expression of genes. The simulation study in the next section uses the following five recently-proposed testing methods. The first two tests, found in the edgeR and DESeq packages, extend Fisher’s exact test to data following negative binomial distribution. The next three tests, developed by Lund, Nettleton, McCarthy, and Smyth [15], are implemented in the QuasiSeq R package, available from the Comprehensive R Archive Network (CRAN). The QL test in the QuasiSeq package uses a quasi-negative binomial model in which a quasi-likelihood dispersion, 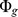, is assigned to each gene (separately from the negative binomial dispersion, 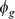) for additional flexibility. The QL test executes a quasi-likelihood ratio test for the differential expression of each gene. The QLShrink test improves on the QL test by borrowing information across genes to estimate the 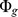
’s. The QLSpline test extends the QLShrink test by using a spline fit to account for the relationship between the 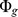
’s and the gene-wise means in the data.

### The Simulation Study

This article presents a simulation study that puts the featured dispersion estimation methods to the test. We first generate pseudo-datasets for which the true negative binomial dispersion parameters and truly differentially expressed genes are known. Then, we apply the featured dispersion estimation methods and testing methods to the pseudo-data, compare the results to the truth, and measure the performance of the dispersion estimation methods in terms of point estimation and performance in testing for differential expression.

#### The underlying real datasets

The simulation study uses two real RNA-seq datasets to generate the pseudo-datasets. The “Pickrell dataset” (Gene Expression Omnibus accession number GSE19480) comes from a study by Pickrell, Marioni, Pai, Degner, Engelhardt, et al. [16], who studied 69 lymphoblastoid cell lines derived from unrelated Nigerian individuals who were subjects in the International HapMap Project. The “Hammer dataset” (Gene Expression Omnibus accession number GSE20895) comes from a study by Hammer, Banck, Amberg, Wang, Petznick, et al. [17], who compared gene expression in the L4 dorsal root ganglia of control rats with those in rats with experimentally induced chronic neuropathic pain. Both of these datasets are publicly available at the Recount database at http://bowtie-bio.sourceforge.net/recount/ [18].

**Figure 1:**
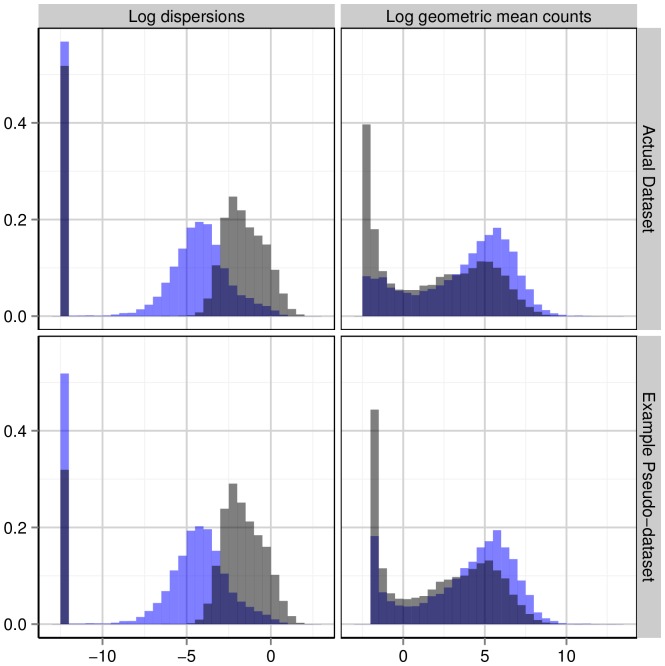
A look at the data. Hammer data and Hammer-generated pseudo-data are in blue, while Pickrell data and Pickrell-generated pseudo-data are shown in black. The top two panels show the gene-wise log geometric mean counts and log dispersion estimates, estimated with the QL method, for the Hammer and Pickrell datasets. The bottom two panels plot the analogous quantities for example simulated pseudo-datasets, except that the log dispersions plotted are the true dispersions used to simulate the pseudo-counts and the gene-wise log geometric mean counts are the 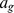
’s (log geometric mean counts from the real data) used in the simulations. The vertical bar at around 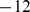 in the plots of the log dispersions is an artifact of the QL method, which sets extremely low dispersions (i.e., dispersions of non-overdispersed genes) to a common minimum value.

**Figure 2:**
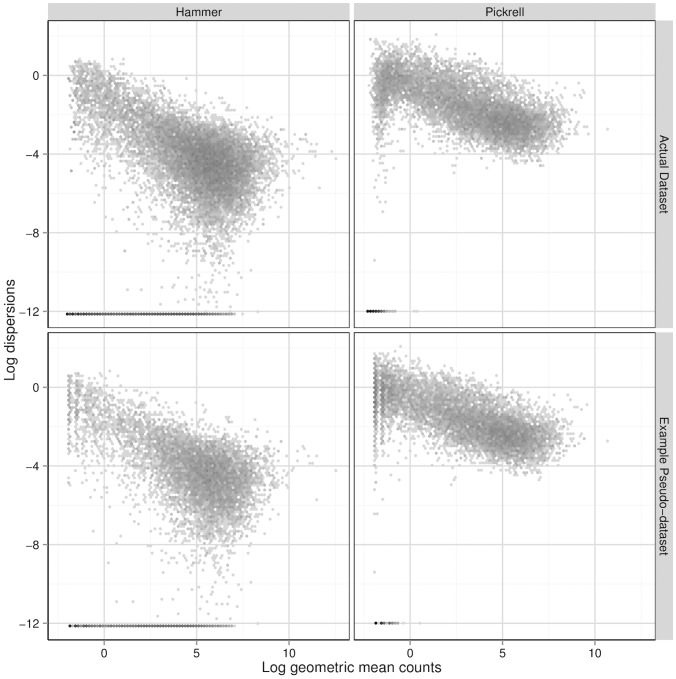
Dispersion-mean relationships. The top two panels show the relationship between the log QL-method-estimated dispersions and the gene-wise log geometric mean counts of the Hammer and Pickrell datasets. The bottom two plot the analogous quantities for example simulated pseudo-datasets, except that the log dispersions plotted are the true log dispersions used to simulate the pseudo-counts (i.e., the 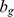
’s) and the gene-wise log geometric mean counts are the 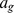
’s used in the simulations. Bins in these two-dimensional histograms are shaded by their log frequency.

The top two panels of Figure 1 show the gene-wise log geometric mean counts and log dispersion estimates, estimated with the QL method, of each of the datasets. The Hammer dataset has higher mean counts and lower dispersions relative to the Pickrell dataset. Hence, tests for differential expression will, in general, have higher power when applied to the Hammer-generated pseudo-datasets than when applied to the Pickrell-generated pseudo-datasets. See the top two panels of Figure 2 for the relationship between the log quasi-likelihood dispersions and the gene-wise log geometric mean counts.

#### Generating a pseudo-dataset

For each one of the real datasets (Hammer or Pickrell), we first compute the two quantities, 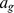 and 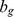, for each gene 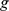, where 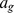 is the geometric mean and 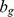 is the dispersion parameter estimated with the quasi-likelihood (QL) method. (All zero read counts are set to a small constant for the geometric mean calculation.) Then, we generate each pseudo-dataset with 10,000 genes as follows:

1. Randomly select 10,000 genes from one of the real datasets (Hammer or Pickrell) without replacement. The corresponding 10,000 pairs of 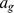 and 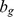 will be used as the the geometric mean expression level across treatments and true dispersion, respectively, of simulated gene 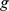.
2. Randomly select simulated gene 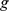 to be either differentially expressed across the two treatments or equivalently expressed such that exactly 20% of the simulated genes are differentially expressed and the remaining 80% are equivalently expressed.
3. Set the log fold change across treatment levels, 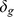, to be zero for all equivalently expressed genes. In order to build a correlation structure into the differential expression pattern of the simulated genes, we draw the 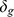
’s of all differentially expressed genes from a multivariate normal distribution with mean 0 and a block-diagonal variance-covariance matrix. Each of the 40 blocks is a 50
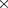
50 correlation matrix randomly drawn from a uniform distribution on the space of all possible 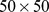 correlation matrices. The study in this article used the rcorrmatrix() function in the ClusterGeneration R package to calculate the correlation matrices. Please see the reference by Joe [19] for the algorithm behind rcorrmatrix().
4. Compute the true mean expression level 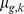 of simulated gene g for treatment levels 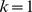 and 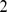 using
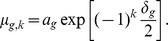

5. Randomly draw the pseudo-count of each simulated gene 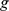 in library 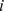 from a NB(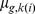, 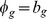) distribution, where 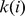 is the treatment group of library 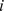.
6. Each gene in the pseudo-dataset should have at least one read to be included in the following analysis. Hence, if the pseudo-counts of simulated gene 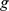 are all zero, we keep 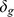 and redraw 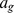 and 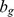, and then redraw the pseudo-counts as in steps 4 and 5.

Note that our method of choosing true mean and dispersion pairs builds an empirical dispersion-mean relationship into the simulated data. As Figures 1 and 2 show, the pseudo-datasets match the real datasets from which they were generated in terms of the distribution of the log gene-wise geometric mean counts, the distribution of the log dispersions, and the dispersion-mean relationship. In step 3, we simulate correlated differentially expressed genes because we expect some differentially expressed genes are dependent in real dataset. We also simulated datasets with 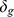
’s from independent standard normal distributions, and the simulation results do not change significantly from what we present later in this article.

Simulation Code S1, the R code used to generate the pseudo-datasets and conduct the analyses, is available as supporting information. The code requires R version 2.15.3 and the R packages listed in Table 1.

**Table 1:** R Packages Required for the Simulation Study’s Implementation.

Package	Version	Repository
abind	1.4–0	CRAN
AMAP.Seq	1.0	CRAN
Biobase	2.18.0	Bioconductor
clusterGeneration	1.3.1	CRAN
DESeq	1.10.1	Bioconductor
DSS	1.0.0	Bioconductor
edgeR	3.0.8	Bioconductor
ggplot2	0.9.3.1	CRAN
hexbin	1.26.1	CRAN
iterators	1.0.6	CRAN
magic	1.5–4	CRAN
MASS	7.3–23	CRAN
multicore	0.1–7	CRAN
plyr	1.8	CRAN
QuasiSeq	1.0–2	CRAN
pracma	1.4.5	CRAN
reshape2	1.2.2	CRAN

For the full implementation, please see Simulation Code S1.

#### Simulation settings

Some pseudo-datasets were generated from the Hammer dataset, while others were generated from the Pickrell dataset. In addition, the number of libraries per treatment group varied from pseudo-dataset to pseudo-dataset. Hence, six “simulation settings”, given in Table 2, were used. 30 pseudo-datasets were generated under each simulation setting.

**Table 2:** Simulation Settings.

Setting	Dataset	Group 1 Libraries	Group 2 Libraries
I	Pickrell	3	3
II	Pickrell	3	15
III	Pickrell	9	9
IV	Hammer	3	3
V	Hammer	3	16
VI	Hammer	9	9

See the end of the Methods section for details.

#### Normalization

Our simulation procedure was configured such that within each pseudo-dataset, the library sizes do not vary systematically. So when analyzing the simulated data, it would be reasonable to set all the library-wise normalization factors, 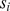, equal to 1. However, since practitioners use nontrivial normalization methods in the field, we borrowed a sophisticated normalization method: specifically, the one described by Anders and Huber [5]. As shown in unpublished work by Xiong and Liu [20], this popular normalization method performs on par with the ubiquitous 0.75 quantile and TMM methods described in the introduction, sometimes even surpassing these two alternatives. The results presented in this article were obtained using the method by Anders and Huber, and they agree with the results obtained from setting all normalization factors equal to 1.

Anders and Huber’s method assigns each library-wise normalization factor, 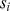, to
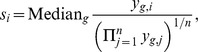
where 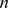 is the total number of libraries. This article uses the implementation in Anders and Huber’s DESeq package, which avoids dividing by zero by skipping genes whose geometric means, 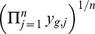, are zero.

The methods implemented in edgeR 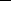 the wqCML and APL dispersion estimation methods and an exact test for differential expression 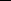 use adjusted library sizes instead of normalization factors. We borrow from the 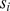
’s computed with DESeq to calculate these adjusted library sizes,
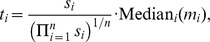
where 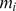 is observed size of library 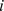.

## Results

With 30 pseudo-datasets generated for each of 6 simulation settings, we apply each dispersion estimation method to each pseudo-dataset, and we use the dispersion estimates to test for the differential expression of genes. We use the true dispersions, the knowledge of which genes are truly differentially expressed, the dispersion estimates, and the test results to compare the dispersion estimation methods. We assess the quality of the methods in terms of the accuracy and precision of point estimation and performance in tests for differential expression.

### Point Estimation

The overall quality of any point estimator can be measured in terms of its mean squared error. For each pseudo-dataset and each dispersion estimation method, we calculate the mean squared error of the transformed estimated dispersions,
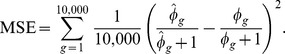



Here, the 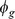
’s are the true dispersions, and the 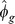
’s are estimates computed with one of the methods described previously. The idea of transforming the dispersions by 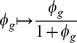, which improves the robustness of MSE to the presence of outliers, was taken from an article by Robinson and Smyth [11].

**Figure 3:**
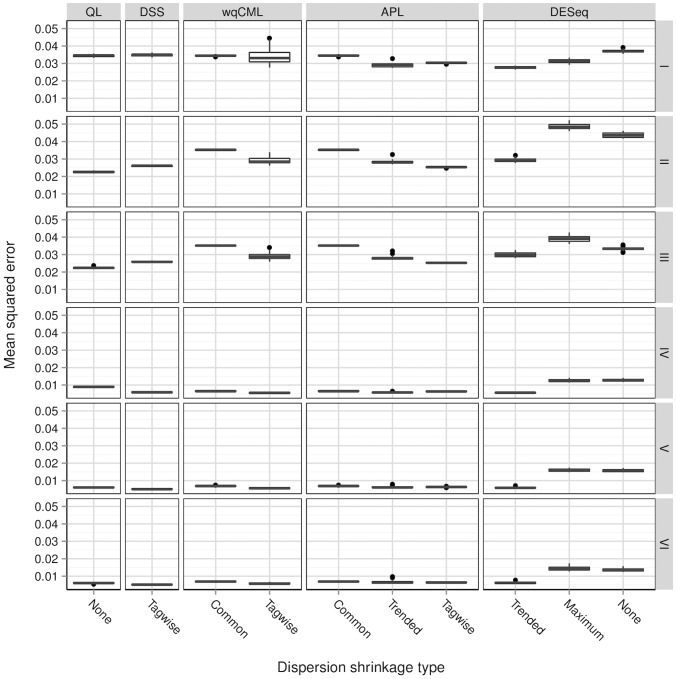
Mean squared error of the transformed dispersions.

Figure 3 displays the MSEs according to dispersion estimation method and simulation setting. The columns correspond to different dispersion estimation methods, and the rows correspond to different simulation settings. There are several types of dispersion shrinkage methods, which are indicated by the labels at the bottom of the figure. The “None” type indicates no shrinkage at all, which means dispersion parameters are estimated for each gene separately. “Common” denotes the methods that give all genes the same estimated dispersion parameter. “Trended” indicates the methods that fit each parameter to a common trend between dispersion and mean expression. “Maximum” refers to the variation of the DESeq method that effectively takes the maximum of the no-shrinkage estimate and the one obtained from the analogous “Trended” option. Lastly, “Tagwise” denotes the methods besides the DESeq “Maximum” method with a moderate level of shrinkage.

Wu, Wang, and Wu [7] show a similar figure (their Figure 3) for the Tagwise wqCML method (called “edgeR” in their paper), the DESeq Maximum method, and the DSS method under their two simulation settings. Their figure suggests that under the MSE metric, the DSS method performs better than the Tagwise wqCML method, which in turn performs better than the DESeq Maximum method. Our results from simulation settings II though VI do not contradict this result. However, in simulation setting I, where sample sizes are extremely small, gene-specific mean counts are relatively low, and dispersions are relatively high (see Figure 1), this ranking is reversed.

Overall, the results for the Hammer-generated pseudo-datasets (simulation settings IV-VI) naturally group the dispersion estimation methods into three categories. The first group includes Maximum DESeq and the no-shrinkage DESeq methods. These methods produce the largest MSE, which is not surprising because the Maximum DESeq method is conservative and designed to obtain larger dispersion and because the no-shrinkage DESeq method applies a naive dispersion estimation technique for each gene independently. The next category includes the QL method alone, which performs better than the Maximum and no-shrinkage DESeq methods, but worse than others when the number of libraries is small (simulation setting IV). The other methods all perform similarly and form a group of MSE-best methods. This demonstrates that shrinkage indeed helps improve the point estimators by borrowing information across genes. However, too much shrinkage is detrimental, as the Common methods perform slightly worse than their Trended and Tagwise counterparts.

Parameter estimation is more challenging for the Pickrell-generated pseudo-datasets (simulation settings I–III) than the Hammer-generated pseudo-datasets (simulation settings IV–VI) because the counts are lower, dispersion is larger in general (Figure 1), and dispersion parameters have a wider spread. (Please note that Figure 1 uses a log scale for the horizontal axis.) MSEs are far greater for settings I–III than for settings IV–VI. In simulation setting I, where the number of libraries is small, the Trended APL, Tagwise APL, and Trended DESeq methods form a group of MSE-best methods. When the number of libraries increases, the DSS and Tagwise wqCML methods also perform well. Interestingly, the “Common” methods underperform, but the QL method is relatively good within settings II and III, which have larger sample sizes than simulation setting I. For these extremely varied dispersions (with a spread of 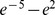 observed in Figure 1), shrinking them toward a common value is not as good as estimating them separately. In all cases, the moderate shrinkage methods are never the worst methods and are often among the best ones.

**Figure 4:**
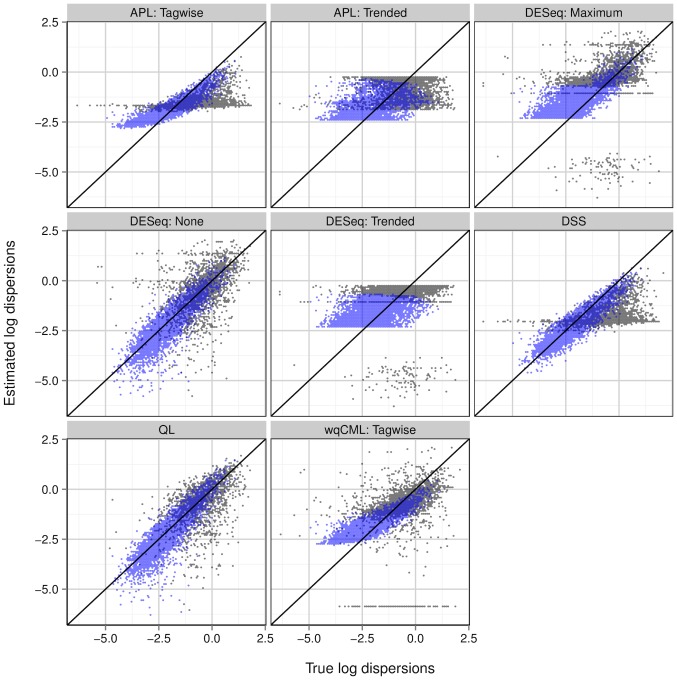
Simulation setting II: estimated vs true dispersions for an example pseudo-dataset. Dispersions with gene-wise log geometric mean counts below the median (log mean from 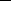
2.17 to 1.63) are shown in black, while those above the median (log mean from 1.63 to 10.6) are shown in light blue. Overlapping points are shown in dark blue. Results for simulation settings I and III are similar.

**Figure 5:**
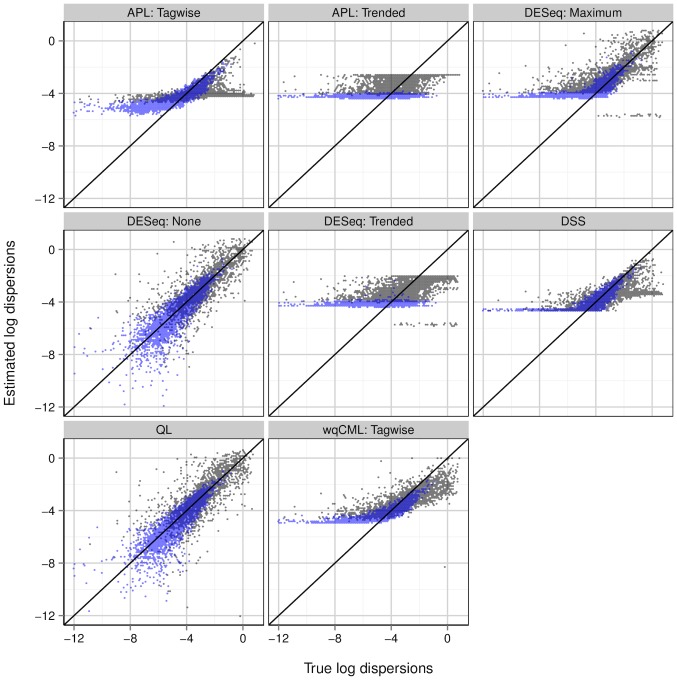
Simulation setting V: estimated vs true dispersions for an example pseudo-dataset. Dispersions with gene-wise log geometric mean counts below the median (log mean from −2.17 to 4.49) are shown in black, while those above the median (log mean from 4.49 to 12.3) are shown in light blue. Overlapping points are shown in dark blue. Results for simulations IV and VI are similar.

Although useful for determining the overall quality of a point estimator, the MSE heuristic is only a single scalar computed for an entire dataset. It is also important to consider the way that estimation error varies with the magnitude of the true dispersions. Figures 4 and 5 plot the log estimated dispersions on the log true dispersions for several dispersion estimation methods for an example pseudo-dataset within each of simulation settings II and V. Analogous plots for simulation settings I and III are similar to Figure 4, and analogous plots for simulation settings IV and VI are similar to Figure 5.

Wu, Wang, and Wu [7] show a similar figure (their Figure 1) for the Tagwise wqCML method (called “edgeR” in their paper), the DESeq Maximum method, and the DSS method. The patterns in their figure approximately agree with our results for simulation settings I through III. However, for simulations IV through VI, our scatterplots of the DSS-estimated dispersions on the true dispersions show a lower truncation that is not present in the figure by Wu, Wang, and Wu. The DSS method may shrink dispersions particularly aggressively under these conditions.

According to Figures 4 and 5, the methods with the least shrinkage 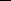 i.e., the QL and no-shrinkage DESeq methods 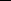 exhibit the widest vertical spread about the identity line, but have patterns most closely resembling this line. Compared with the other methods, estimation error is high for these no-shrinkage methods, but maximally correlated with true dispersion magnitude. On the other hand, both of the trended methods (Trended APL and Trended DESeq) show the lowest vertical spreads, but the greatest systematic departures from the identity line. These trended methods systematically underestimate large true dispersions and systematically overestimate small true dispersions, even placing sharp upper and lower bounds on most of the estimated dispersions. Interestingly, the dispersions estimated with the moderate-shrinkage methods (DSS, Tagwise wqCML, Tagwise APL, and Maximum DESeq) show relatively high agreement with the true dispersions for simulation settings I-III (Pickrell-generated pseudo-data) but much lower agreement with the low true dispersions in simulation settings IV-VI (Hammer-generated pseudo-data). In practice, this reluctance to produce small dispersions may mitigate the false detection of differentially expressed genes.

Figures 4 and 5 show that some methods systematically overestimate low true dispersions and systematically underestimate high true dispersions. This behavior is exactly what we should expect from shrinkage. Intuitively, shrinkage pulls estimates towards some common focal point. As an immediate and important practical consequence for point estimation, low estimates should increase, high estimates should decrease, and the collective scatter of estimates should narrow. The practical consequences of shrinkage for significance testing, however, are not clear from Figures 4 and 5 alone. For real insight into hypothesis testing, we need the results of the next subsection.

### Test Performance

Since the detection of differentially expressed genes is the major goal of most RNA-seq experiments, it is vitally important to measure and compare the direct impact of the dispersion estimation methods on the detection of differentially expressed genes, which is why the pseudo-datasets are generated such that each simulated gene is known to be either differentially expressed or equivalently expressed. Using this knowledge and the p-values obtained from the tests for differential expression, a receiver operating characteristic (ROC) curve is constructed for each pseudo-dataset/test for differential expression/dispersion estimation method combination.

An ROC curve is a graph of the true positive rate (TPR) of the detection of differentially expressed genes vs the false positive rate (FPR). In practice, we define FPR and TPR to be functions of the significance level, 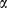, of the tests for differential expression. Specifically,
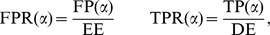
where FP(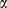) and TP(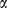), respectively, are the numbers of false positive and true positive detections at significance level 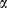. Also, EE and DE, respectively, are the true numbers of equivalently expressed and differentially expressed genes. In the pseudo-data, we know that EE is 8000 and DE is 2000. In fact, we know exactly which genes are differentially expressed, so we can calculate FP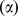 to be the number of equivalently expressed genes with p-values less than 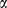 and TP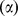 to be the number of differentially expressed genes with p-values less than 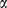. Using a fixed range of 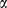 values (the same for all ROC curves in this study), we calculate multiple points of the form, (FPR(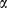), TPR(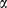)) and plot them on the x-y plane. To compensate for gaps in the x direction of the graph, we interpolate the points with a step function that lies beneath a simple linear interpolation, the latter of which may artificially inflate the AUC heuristic explained below.

**Figure 6:**
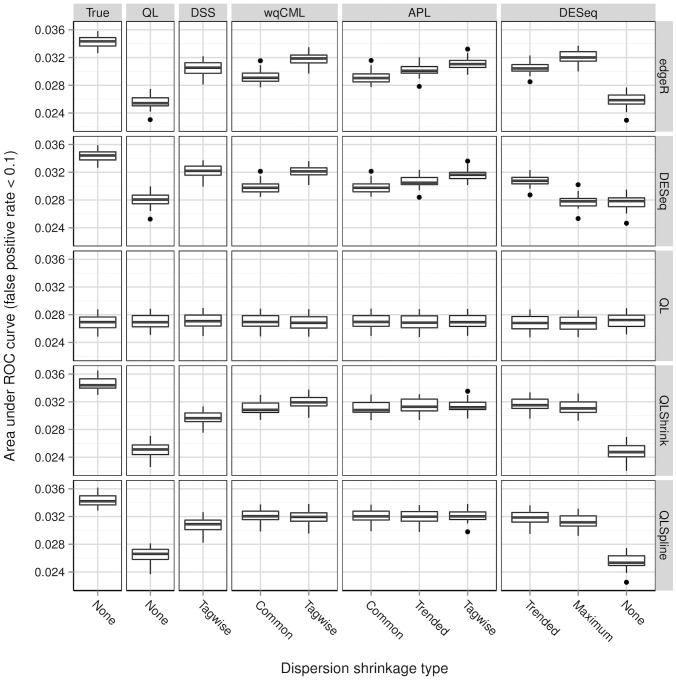
Simulation setting I: areas under ROC curves. Boxplots of AUC calculated based on 30 pseudo-datasets are shown for each combination of dispersion estimation method and test for differential expression.

In this study, we use the area under each ROC curve (AUC) as a relative measure of the quality of a test, where a high AUC indicates relatively good test performance. Here, each AUC is computed only for FPR 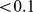 so that testing situations are evaluated only at reasonable significance levels. Please note that AUC not only depends on the quality of a test, but also on the magnitude of differential expression. In Figures 6 through 11, the AUC values are small because in our simulations, the true log fold changes, 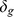, were taken from a standard normal distribution. As a result, the magnitude of differential expression was small for a significant fraction of truly differentially expressed genes.

**Figure 7:**
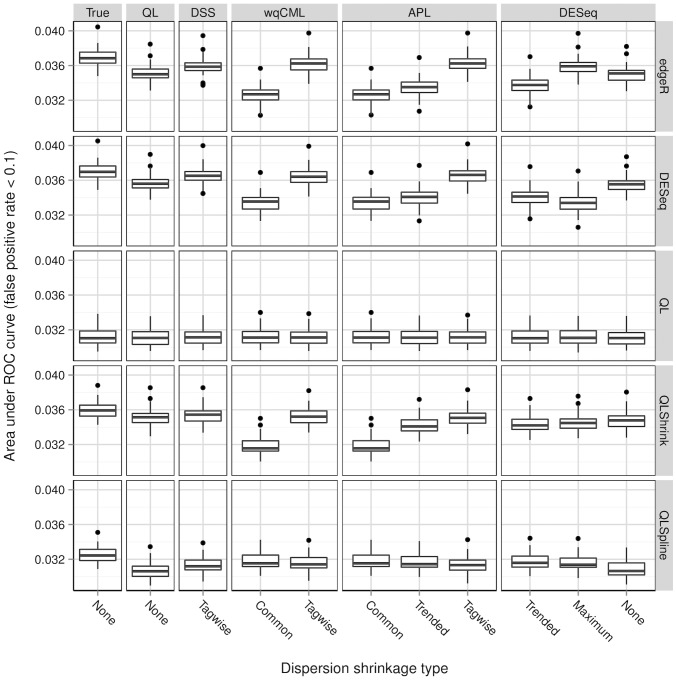
Simulation setting II: areas under ROC curves. Boxplots of AUC calculated based on 30 pseudo-datasets are shown for each combination of dispersion estimation method and test for differential expression.

**Figure 8:**
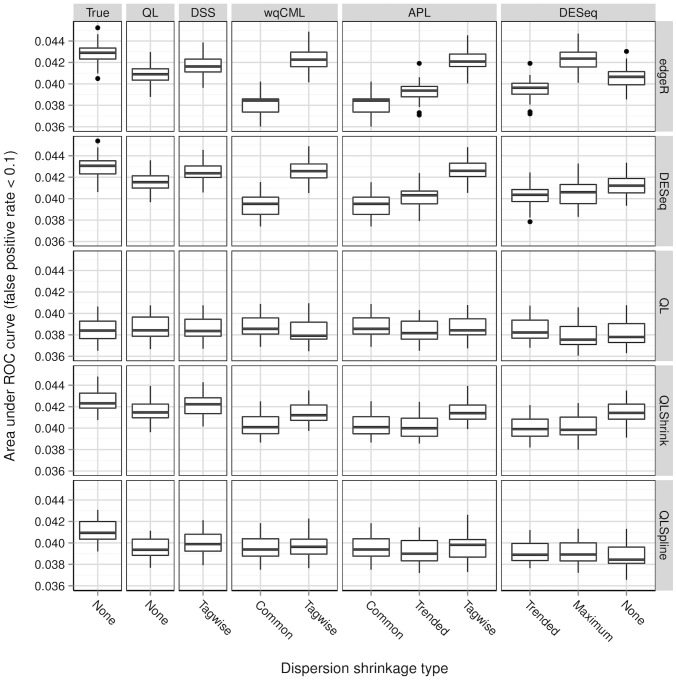
Simulation setting III: areas under ROC curves. Boxplots of AUC calculated based on 30 pseudo-datasets are shown for each combination of dispersion estimation method and test for differential expression.

**Figure 9:**
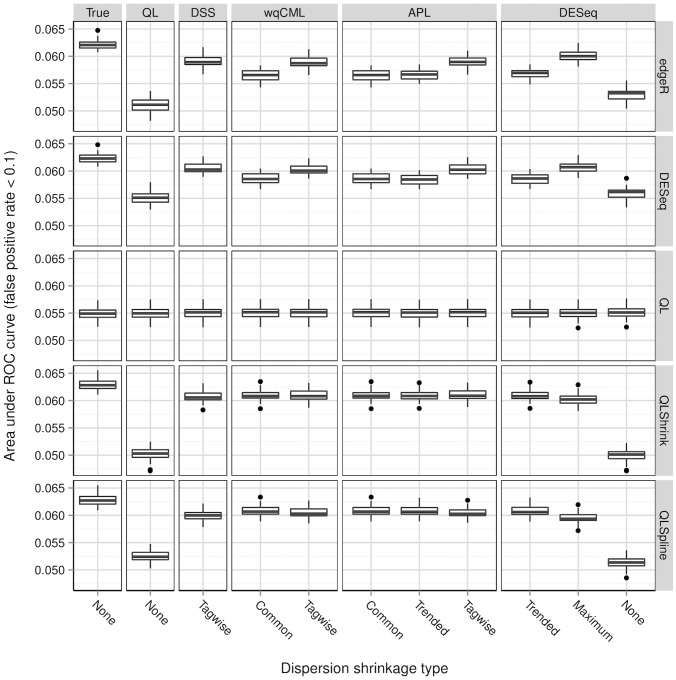
Simulation setting IV: areas under ROC curves. Boxplots of AUC calculated based on 30 pseudo-datasets are shown for each combination of dispersion estimation method and test for differential expression.

**Figure 10:**
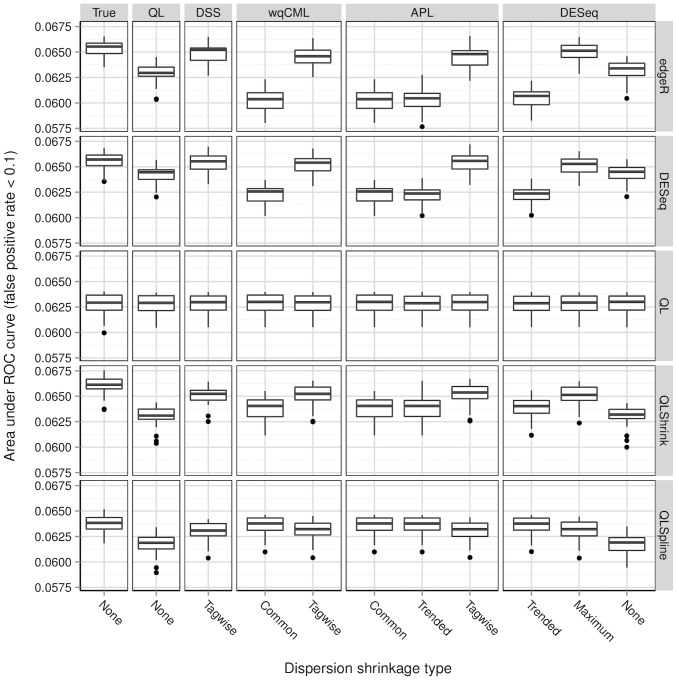
Simulation setting V: areas under ROC curves. Boxplots of AUC calculated based on 30 pseudo-datasets are shown for each combination of dispersion estimation method and test for differential expression.

**Figure 11:**
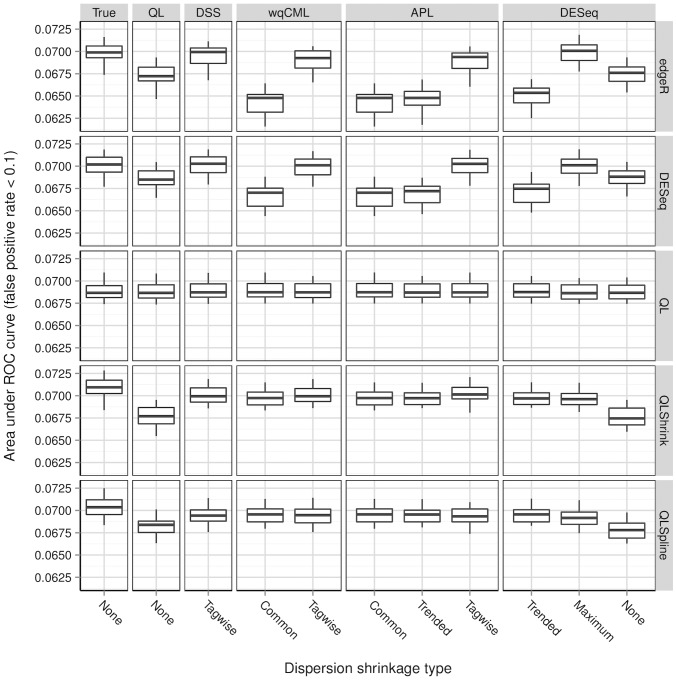
Simulation setting VI: areas under ROC curves. Boxplots of AUC calculated based on 30 pseudo-datasets are shown for each combination of dispersion estimation method and test for differential expression.

Figures 6, 7, 8, 9, 10, and 11 show the relationships between AUC and dispersion estimation method for each test setting and simulation setting. We include the results of tests using the true dispersion parameters and use these results as the “gold standard” to evaluate all dispersion parameter estimation methods.

Table 2 of the paper by Yu, Huber, and Vitek [8] suggests that the Tagwise wqCML method (called “edgeR” in their paper) and the DESeq dispersion estimation method perform roughly equally well under the AUC metric. Our results agree, with the exception of the DESeq testing method in simulation settings I through III, where the DESeq dispersion estimation method performs worse than the Tagwise wqCML method.

Overall, the three tests in the QuasiSeq package are less affected by dispersion estimation than the edgeR and DESeq exact tests. These tests introduce gene-wise quasi-likelihood dispersion parameters to the negative binomial model, and the new parameters absorb some of the variability that would otherwise manifest solely in the negative binomial dispersions. The practical upshot is that all three QuasiSeq tests are relatively robust under both noisy data and poor estimation of negative binomial dispersions. The QL test is an extreme case, with little change in its AUC boxplots across the dispersion estimation methods, because it does not apply any special constraints to the quasi-likelihood dispersions. (On the other hand, the QLShrink test shrinks the quasi-likelihood dispersions using a common value, and the QLSpline test shrinks them using a fitted spline.) Unfortunately, the QL test also performs the worst among the five tests overall, making it a poor choice in practice despite its otherwise useful robustness. The QLSpline test is better than the QL test, and the QLShrink test is better still.

The rankings of the dispersion estimation methods are similar among the edgeR exact test, the DESeq exact test, and QLShrink test. Specifically, the DSS, Tagwise wqCML, and Tagwise APL – i.e., the moderate-shrinkage methods – are the best. Not only do these dispersion estimation methods perform well relative to other methods, but they are also extremely close to the true dispersions in terms of AUC. Methods with an extremely large or extremely small degree of dispersion shrinkage – i.e., the Trended, Common, and “None” modes of dispersion shrinkage – are subject to relatively poor performance in at least one of the simulation settings. Interestingly, the Maximum DESeq method also performs well in terms of AUC in several cases, although it does poorly with respect to MSE. This shows that accurate and precise estimation of dispersions and optimal test performance do not always go together.

Across all six simulation settings, when combined with moderate-shrinkage methods for dispersion, the best tests for differential expression are the edgeR exact test, the DESeq exact test, and the QLShrink test. These methods for testing perform roughly equally well, and they are better than other combinations of tests and dispersion estimation methods. In some cases, this difference in AUC between the two tiers of tests is dramatic. With the addition of the gene-wise quasi-likelihood dispersion parameter to the negative binomial model, the QLShrink test is more robust to changes in dispersion estimation method than the either of the exact tests, which is most noticeable for the Pickrell-generated pseudo-datasets (simulation settings I-III). In practice, we recommend using the QLShrink test because we expect the addition of quasi-likelihood dispersion parameters to make the QLShrink test more flexible than the edgeR and DESeq exact tests under departures from the negative binomial model.

## Discussion

It is challenging to estimate negative binomial dispersions from RNA-seq data due to the “small 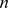, large 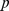
” problem. Methods that borrow information across genes are better than methods that estimate parameters independently for each gene. If we assume that the dispersion parameters are the same for all genes, then we can use the entire dataset to compute a precise estimate of a shared dispersion parameter. However, assuming a common dispersion for all genes is too unrealistic in practice. For example, the dispersion estimates for the the Pickrell dataset computed with the QL method range from about 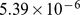 to about 8.35. We expected the optimal dispersion estimation methods to instead use a moderate degree of shrinkage: that is, to “borrow information” across genes, using the whole dataset to compute a common value, trend, or prior distribution for the dispersions, and then shrink individual gene-wise dispersion estimates toward this chosen anchor. Indeed, our simulation results show that the moderate-shrinkage methods – the DSS, Tagwise wqCML, and Tagwise APL methods – perform relatively well in terms of MSE and optimally in terms of the performance of tests for differential expression. Although DSS shrinks the dispersions towards a common prior, Tagwise wqCML shrinks them towards a common value, and Tagwise APL uses neighboring genes on a common trend for shrinkage, these three optimal methods perform roughly equally well in our simulations. Thus, the degree of dispersion shrinkage is more important than how this shrinkage is achieved.

These moderate shrinkage methods outperform the others in all five featured tests for differential expression. However, these tests do not perform equally well. The edgeR exact test, DESeq exact test, and QLShrink test outperform the other two. Furthermore, the performances of the edgeR and DESeq tests depend highly on the dispersion estimation method chosen, while the addition of a gene-wise quasi-likelihood dispersion parameter gives the QLShrink test extra robustness under the choice of dispersion estimation method. We expect this same flexibility to help the QLShrink test perform especially well under departures from the negative binomial model, so we recommend using the QLShrink method with either the DSS, Tagwise wqCML, or Tagwise APL method in practice.

Interestingly, the ranking of dispersion estimation methods according to MSE is not the same as the ranking according to AUC. A notable example is the Maximum DESeq method, which performs poorly in terms of MSE, but often performs extremely well in tests for differential expression. This behavior may result from the intentional overestimation of the dispersions, which contributes to a high MSE, but guards against false positives. In addition, methods with similar MSE may have very different AUC. For example, the Trended APL and Tagwise APL methods yield similar MSEs in simulation setting V, but the Tagwise APL method performs much better than the Trended APL method in the edgeR test (Figure 10). We do not have an explanation for this behavior, but we advise practitioners to think about point estimation and test performance separately when estimating negative binomial dispersions.

## Supporting Information

Simulation Code S1 **R code used to generate the pseudo-datasets and conduct the analyses.** The software requirements can be found in Table 1.
(R) Click here for additional data file.

## References

[1] Oshlack A, Robinson MD, Young MD (2010) From rna-seq reads to differential expression results. Genome Biology 11. 10.1186/gb-2010-11-12-220 PMC3046478 21176179

[2] Wang L, Li P, Brutnell TP (2010) Exploring plant transcriptomes using ultra high-throughput sequencing. Briefings in Functional Genomics 9: 118–128. 20130067 10.1093/bfgp/elp057

[3] Cameron AC, Trivedi PK (1998) Regression Analysis of Count Data. Cambridge University Press.

[4] Si Y, Liu P (2012) An optimal test with maximum average power while controlling fdr with application to rna-seq data. Biometrics 69: 594–605. 10.1111/biom.12036 23889143

[5] Anders S, Huber W (2010) Differential expression analysis for sequence count data. Genome Biology 11. 10.1186/gb-2010-11-10-r106 PMC3218662 20979621

[6] Robinson MD, Oshlack A (2010) A scaling normalization method for differential expression analysis of rna-seq data. Genome Biology 11: 1275–1282. 10.1186/gb-2010-11-3-r25 PMC2864565 20196867

[7] Wu H, Wang C, Wu Z (2012) A new shrinkage estimator for dispersion improves differential expression detection in rna-seq data. Biostatistics 1: 1–24. 10.1093/biostatistics/kxs033 PMC3590927 23001152

[8] Yu D, Huber W, Vitek O (2013) Shrinkage estimation of dispersion in negative binomial models for rna-seq experiments with small sample size. Bioinformatics 29: 1275–1282. 23589650 10.1093/bioinformatics/btt143 PMC3654711

[9] Si Y (2012). Package ‘amap.seq’. http://cran.r-project.org/web/packages/AMAP.Seq/AMAP.Seq.pdf.

[10] Robinson MD, Smyth GK (2008) Small-sample estimation of negative binomial dispersion, with applications to sage data. Biostatistics 9: 321–332. 17728317 10.1093/biostatistics/kxm030

[11] Robinson MD, Smyth GK (2007) Moderated statistical tests for assessing differences in tag abundance. Bioinformatics 23: 2881–2887. 17881408 10.1093/bioinformatics/btm453

[12] Robinson MD, McCarthy DJ, Smyth GK (2009) edger: a bioconductor package for differential expression analysis of digital gene expression data. Bioinformatics 26: 139–140. 19910308 10.1093/bioinformatics/btp616 PMC2796818

[13] Robinson MD, McCarthy DJ, Chen Y, Smyth GK (2012). Package ‘edger’. http://www.bioconductor.org/packages/2.10/bioc/manuals/edgeR/man/edgeR.pdf.

[14] McCarthy DJ, Chen Y, Smyth GK (2012) Differential expression analysis of multifactor rna-seq experiments with respect to biological variation. Nucleic Acids Research 40: 4288–97. 22287627 10.1093/nar/gks042 PMC3378882

[15] Lund SP, Nettleton D, McCarthy DJ, Smyth GK (2012) Detecting di_erential expression in rna-sequence data using quasi-likelihood with shrunken dispersion estimates. Statistical Applications in Genetics and Molecular Biology 11. 10.1515/1544-6115.1826 23104842

[16] Pickrell JK, Marioni JC, Pai AA, Degner JF, Engelhardt BE, et al (2010) Understanding mechanisms underlying human gene expression variation with rna sequencing. Nature 464: 768–72. 20220758 10.1038/nature08872 PMC3089435

[17] Hammer P, Banck MS, Amberg R, Wang C, Petznick G, et al (2010) mRNA-seq with agnostic splice site discovery for nervous system transcriptomics tested in chronic pain. Genome Research 20: 847–60. 20452967 10.1101/gr.101204.109 PMC2877581

[18] Langmead B, Frazee A (2012). Recount: A multi-experiment resource of analysis-ready rna-seq gene count datasets. http://bowtie-bio.sourceforge.net/recount/. 10.1186/1471-2105-12-449 PMC3229291 22087737

[19] Joe H (2006) Generating Random Correlation Matrices Based on Partial Correlations. Journal of Multivariate Analysis 97: 2177–2189.

[20] Xiong Y, Liu P (2012) Evaluation of normalization methods for differential expression analysis in RNA-seq experiments. Master of Science creative component, Iowa State University.

